# Influence of Race Performance and Environmental Conditions on Exertional Heat Stroke Prevalence Among Runners Participating in a Warm Weather Road Race

**DOI:** 10.3389/fspor.2019.00042

**Published:** 2019-10-04

**Authors:** Andrew J. Grundstein, Yuri Hosokawa, Douglas J. Casa, Rebecca L. Stearns, John F. Jardine

**Affiliations:** ^1^Department of Geography, University of Georgia, Athens, GA, United States; ^2^Faculty of Sport Sciences, Waseda University, Saitama, Japan; ^3^Korey Stringer Institute, Department of Kinesiology, University of Connecticut, Storrs, CT, United States; ^4^Falmouth Hospital, Falmouth, MA, United States

**Keywords:** road race, exertional heat stroke, wet bulb globe temperature, race medicine, pacing

## Abstract

The New Balance Falmouth Road Race held in Falmouth, Massachusetts, U.S. is a short distance race (11.26 km) that is well-known for high rates of exertional heat stroke (EHS). Previous research has documented the increased EHS rates with hotter and more humid weather conditions, yet did not explore the influence of race pacing on EHS risk. In this study, we leverage 15 years of data to investigate if runners who experienced an EHS moderate their average paces based on weather conditions and if there is a difference in average race pace between participants who experienced an EHS and other runners. Results indicate that runners who experience an EHS do not appear to reduce their average pace with increasing WBGT warning flag categories. In addition, runners who suffer an EHS run at a faster average pace than others, even when controlling for age, gender, race performance, and starting time WBGT. This suggests the important role of metabolic heat production as a risk factor of EHS. Since race pacing is a modifiable risk factor, our findings support the need for race organizers to actively encourage runners to adjust race pacing based on weather conditions.

## Introduction

Exertional heat stroke (EHS) becomes a major concern for race day medical service, especially in warm and hot weather races, where a rapid increase in number of medical tent visits is observed (DeMartini et al., 2014; Hosokawa et al., 2018). EHS occurs when one's thermoregulatory capacity is overwhelmed due to imbalances between metabolic heat production and heat dissipation by means of evaporation, radiation, convection, or conduction (Armstrong et al., 2007; Grundstein et al., 2017). In the context of outdoor exercise in the heat, the rate of rise in internal body temperature is directly influenced by the absolute evaporative requirement needed to maintain heat balance, which is increased as the air temperature, and work output increases (Cramer and Jay, 2016). When the maximum possible evaporative heat loss exceeds the rate of evaporative heat loss required, a state of uncompensable heat stress ensues (Cramer and Jay, 2016). During uncompensable heat stress, the sustained rise in body temperature could lead to cell anoxia, gastrointestinal permeability, cardiovascular collapse, and inflammatory reactions (Epstein and Yanovich, 2019). If the state is not corrected by adjustment (i.e., reduction) in metabolic heat production, the relative risk of exertional heat illness is high since the metabolic rate is the primary driver for heat storage (Santee and Gonzalez, 1998). Notwithstanding, exercise in hot environment (≈34^o^C) has shown to induce an anticipatory reduction in work output without an evidence of neuromuscular fatigue or influence from the rate of heat body heat storage, suggesting that sensory input from warm environment itself may also have influence on exercise pacing (Ravanelli et al., 2014). From these laboratory findings, the difference between runners who experienced EHS and those who did not may be reflected in their pacing strategy. EHS runners may have sustained faster pace exposing themselves to longer uncompensable heat stress while those who did not had attenuated their pace as the environmental temperature increased. Therefore, we aimed to retrospectively examine the influence of pacing on the likelihood of a runner experiencing EHS. We hypothesized that the pace of runners who experience an EHS would differ from non-EHS finishers.

## Materials and Methods

### Study Design

We studied the interrelationship between meteorological conditions, race pacing, and cases of EHS at the New Balance Falmouth Road Race. The race is 11.26 km in length and is held annually in August in Falmouth, Massachusetts, USA (41.52°N, 70.67°W). Each year, ~10,000 runners, ranging in status from novice to elite, participate in the race.

### Race-Participant Data

Data on EHS cases were obtained from medical tent records for 15 years between 2003 and 2018 (no finish times were recorded in 2006). EHS was clinically determined based on a rectal temperature ≥40°C and associated signs of central nervous system dysfunction (e.g., delirium, altered mental status, aggression, hysteria). Only EHS cases where the participant finished the race were included in this analysis. The finish time was obtained for each of these individuals and average pacing was calculated by dividing finish time by race distance (11.26 km). In addition, information on the age, gender, and race status as elite or non-elite were available for both EHS and non-EHS finishers for the years 2014–2018 (New Balance Falmouth Road Race, 2019). In these 5 years, we used the race bib numbers to explicitly identify elite (bib numbers 1–100 for men and 101–200 for women) and non-elite (bib numbers >201) runners. We did not have these data for the years 2003–2013 and instead divided runners as “faster” or “slower” based on available finish times for 2014–2018. “Faster” runners were defined those with finish times ≤ 2 standard deviations from the mean and would have finish times consistent with those of elite runners but also some exceptional non-elite runners. This value ranged from ~42–44 min between years, with an average of 43:33 min. Racers with times greater than the 43:33 min threshold were considered “slower” runners. Our sample of faster EHS runners was insensitive to the particular threshold used (42–44 min).

Lastly, incidence rates were computed as number of EHS finishers per 1000 finishers for the years 2003–2018, with the exception of 2012 and 2013 when the number of finishers were not available. The number of finishers for 2003–2011 were obtained from published results in DeMartini et al. (2014) and for 2014–2018 from the New Balance Falmouth Road Race website (New Balance Falmouth Road Race, 2019).

### Meteorological Data

Meteorological data (e.g., dry bulb temperature, dewpoint temperature, and cloud cover) were collected from the nearest available weather observing station located at Otis Air National Guard Base (41.65°N, −70.52°W), which is ~18.8 km from the race start and operated in joint effort between the National Weather Service, the Federal Aviation Administration, and the Department of Defense. Wet bulb globe temperatures (WBGT) were not routinely recorded during race events and were therefore computed from meteorological data using the Heat Stress Adviser (version 2005; Zunis Foundation, Tulsa, OK) software package (Coyle, 2000). Input data into this model includes air temperature, dewpoint temperature, cloud cover, and time of day. The model was developed for warm season conditions (May—September) and was tested in a variety of geographic regions in the U.S., including Oklahoma, Texas, Minnesota, and New York. WBGT estimates are accurate to ±1.1°C (Coyle, 2000; Zunis Foundation, 2005).

The WBGT was classified using the International Institute for Race Medicine (IIRM) heat stress flag color warnings for runners as low (green, <18°C), moderate (yellow, <18–23°C), high (red, 23–28°C), and extremely high (black, >28°C) risk for hyperthermia (Mears and Watson, 2015). The WBGT was determined at the start of the race (either 9 or 10 a.m. Eastern Daylight Time, depending on the year) and also computed as an average based on the race finish time for each runner suffering an EHS.

### Statistical Analysis

All statistical analyses were performed using SPSS (Version 26; IBM Corporation, Aramonk, NY). The relationship between EHS incidence rates and starting time WBGT was assessed both graphically and using regression analysis. *R*^2^ was used to identify the explained variance of the regression model.

Descriptive statistics (e.g., mean, standard deviation) were used to characterize average race pacing for EHS cases under different flag color warnings. Group differences in race pacing among flag warning categories for faster and slower EHS finishers were determined using one-way ANOVA. Statistical significance was set a priori at an alpha level of ρ <0.05. The Shapiro-Wilk test of normality and Levene's test for equality of variances were used to ensure that conditions of the statistical test were met. For slow EHS finishers, four participants with race finish times over 1:50 were excluded from analysis as these outliers had average paces consistent with walking rather than running.

Finally, a one-way ANCOVA was conducted to compare any difference in average pacing between EHS and non-EHS finishers for the years 2014–2018 while controlling for age, gender, status as elite or non-elite and starting time WBGT. Statistical significance was set a priori at an alpha level of ρ <0.05. Levene's test for equality of variances and normality checks were used to ensure that conditions of the statistical test were met.

## Results

### Incidence

A total of 247 participants finished the race and experienced an EHS (243 when the four walkers were excluded) over the 2003–2018 period (Table 1). We identified 36 faster and 207 slower EHS finishers using race finish time to stratify the groups. The number of total cases ranged from a low of 5 in 2004 to a high of 38 in 2015, with an average number of 16.5 ± 9.7 per year. EHS incidence rates averaged 1.8 ± 1.16 per 1000 runners and ranged from a low of 0.90 in 2018 up to 4.59 in 2003.

**Table 1:** Finisher data and exertional heat stroke incidence and rate per 1,000 runners by year.

**Year**	**Finishers**	**EHS cases**	**EHS rate**
2003	8,058	37	4.59
2004	8,171	5	0.61
2005	7,532	19	2.52
2007	8,926	10	1.12
2008	8,743	15	1.72
2009	8,864	13	1.47
2010	9,653	6	0.62
2011	10,930	13	1.19
2012		11	
2013		15	
2014	11,060	17	1.54
2015	10,691	38	3.55
2016	10,381	16	1.54
2017	10,901	22	2.02
2018	11,063	10	0.90
**Mean**	9613.31	16.47	1.80
**StDev**	1289.74	9.66	1.16

*EHS rates could not be computed for 2012 and 2013 because no finisher data were available*.

### Wet Bulb Globe Temperature

**Figure 1:**
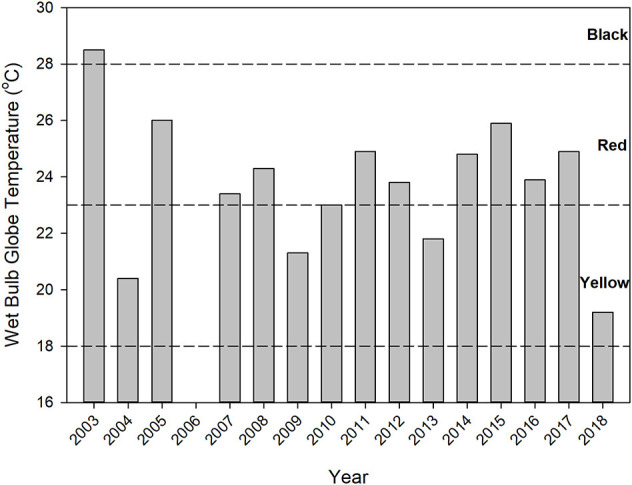
Start time WBGTs and associated flag color warnings indicated by dashed horizontal lines. No finish times were available for EHS cases in 2006 and WBGT data were excluded from the study.

**Figure 2:**
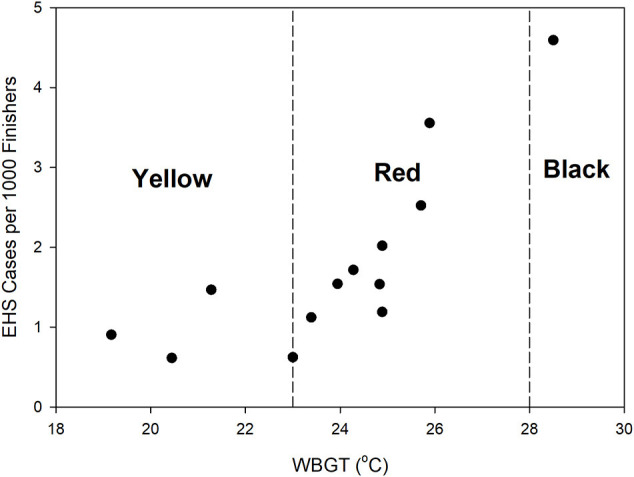
Relationship between start time WBGT and EHS rate per 1000 finishers. IIRM warning flags for runners are categorized as low (green, <18°C), moderate (yellow, <18–23°C), high (red, 23–28°C), and extremely high (black, >28°C) risk for hyperthermia (Mears and Watson, 2015).

WBGTs were computed at the start of the race (Figure 1) and as an average exposure WBGT for each of the 243 runners experiencing an EHS over the length of their race, ranging from ~32–95 min. The average starting time WBGT was 23.7 ± 2.4°C (range = 19.2–28.5°C). The flag warning categories for race start times over the 15 years included 4 yellow (26.7%), 10 red (66.7%), and 1 black (6.7%) (Figure 1). Start time flag warning categories provided a conservative estimate of conditions over the length of the race. In only 1 year (2010) did a flag category shift when considering the average exposure WBGT, and this involved a small decrease in WBGT that shifted the category from red (23.0°C) to yellow (22.6°C). Thus, we used the start time WBGT in additional analyses. The EHS rate per 1000 finishers was plotted against the start time WBGT (Figure 2). As the number of finishers were not available in 2012 and 2013, EHS rates were calculated for the remaining 13 years. We observed that EHS rates increased with increasing start time WBGT, with an *r*^2^ = 0.61 (*p* = 0.001) (Figure 2).

### Race Pace per WBGT Flag Color Warning

**Figure 3:**
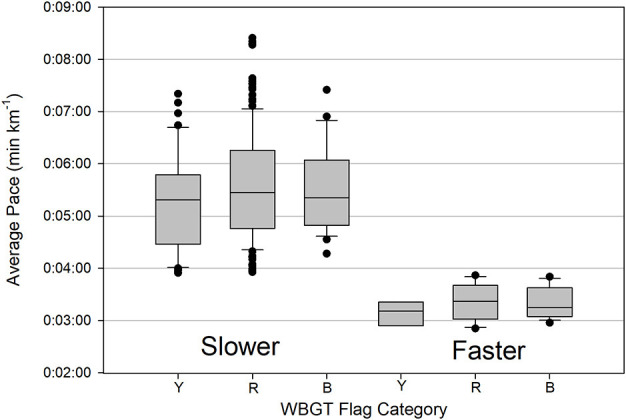
Box and whisker plot of average race pacing among IIRM WBGT warning flag categories (Y is yellow, R is red, and B is black) for faster and slower EHS finishers. The dark line in the box is the median, the top and bottom of the box are 75 and 25 percentiles, respectively, the top and bottom lines are the 90th and 10th percentiles, respectively, and the circles represent outliers.

Average running paces for 243 EHS finishers were compared based on WBGT warning flag category (Figure 3). Faster EHS finishers has average race paces ranging from 3:09 to 3:21 min km^−1^ compared with average paces between of 5:15 to 5:34 min km^−1^ for slower EHS finishers. We found insufficient evidence to indicate a statistically significant difference in pacing among flag warning categories for either faster (*p* = 0.555) or slower runners (*p* = 0.227).

### Race Finish Times for EHS Finishers vs. Overall Runner Population

The average running paces for both EHS and non-EHS finishers (elite and non-elite) were compared for the years 2014–2018. During this period, there were 53,369 non-EHS finishers and 100 EHS finishers (93 non-elite and 7 elite). There is a significant difference in average racing pacing between EHS and non-EHS runners, when controlling for age, gender, race performance (fast vs. slow), and starting time WBGT [*F*_(1, 53, 444)_ = 44.120, *p* < 0.0001). Indeed, the estimated marginal means indicates the average running pace was faster for EHS finishers (5:35 min km^−1^) compared with non-EHS finishers (6:18 min km^−1^).

## Discussion and Conclusions

The purpose of this study was to identify if pacing played a role in the incidence of EHS at the New Balance Falmouth Road Race. This race provides an excellent case study because of the high rates of EHS compared with other road races (DeMartini et al., 2014). Thermoregulation during exercise in heat is influenced by the amount of metabolic heat produced and the amount of heat that is effectively dissipated through some combination of evaporation, radiation, convection, or conduction, depending on environmental conditions. Previously, DeMartini et al. (2014) examined the relationship between environmental conditions and the incidence of EHS. They reported greater incidence of EHS as the environmental condition become warmer. This outcome shows that the margin for amount of heat dissipation (i.e., allowed-heat dissipation) becomes narrower as the environmental condition becomes warmer, making it harder for runners to meet the heat dissipation required to maintain thermoregulation. Previous studies did not, however, address the role of race pacing among EHS runners, which is directly associated with the extent of metabolic heat produced by runners.

It has been speculated that the unusually high EHS rates at Falmouth are related to not only the warm conditions but also the high intensity and greater metabolic heat production produced in this shorter (11.26 km) race compared to longer marathons (DeMartini et al., 2014; Adams et al., 2016). The running speed has been strongly correlated with rises in core body temperatures (e.g., Noakes et al., 1991; Cheuvront and Haymes, 2001; Roberts, 2006) during races. Theoretically, runners should self-regulate their pacing strategy based on some combination of physiological feedback and previous experience (Tucker and Noakes, 2009). This often does happen as decrements in performance are observed in increasingly warm and hot conditions (e.g., Zhang et al., 1992; McCann and Adams, 1997; Ely et al., 2007). Roberts (2006) even observed an EHS suffered under mild weather conditions (ambient temperature 9.5°C) caused by the runner's fast pace in the last 16 km of a race, which suggests that sustained fast pace running can increase the relative risk of EHS even when the environmental conditions seem favorable from human heat balance standpoint. Our findings lend support to these conclusions. We observed that the average pace of EHS finishers was greater than non-EHS finishers, even when controlling for age, gender, race performance (elite or non-elite), and WBGT. A plausible explanation for this association is that having a faster average pace would result in relatively greater metabolic heat production and a greater chance of reaching the upper limit of thermoregulatory threshold that could increase the relative risk of EHS. Since etiology of EHS is multifactorial, we acknowledge that faster average pace alone would not induce elite or fitter individuals to collapse to EHS. However, because pacing is one of the few modifiable risk factors that these runners may consciously adjust at their own discretion, educational efforts should be made by race organizers to prevent overzealous runners from trying to achieve a goal finish time that is not safe at a given environmental condition.

Given that these runners competing at faster average paces may be at higher relative risk for EHS, mitigation measures should be attempted. In athletic settings, there are often external motivation and peer pressure (i.e., competition against teammates, desire to impress peer, a pre-set goal to achieve a personal best) that may push the athletes to continue exercise at an intensity that is unmatched to their physical fitness (Adams et al., 2016). Such external drive to sustain physical effort unmatched to fitness is one of the key risk factors of EHS (Rav-Acha et al., 2004). Although this is an inherent trait of any athlete who has a set goal, runners that participate in race events must first recognize and understand the importance of self-pacing and adjusting it accordingly to the environmental condition. At present, the Falmouth Road Race does not post start time WBGT values to provide guidance for runners. Informing runners of weather conditions to help guide them to optimizing their performance for safety might be a simple and cost-effective approach. One approach prior to the race would be to send informational e-mails to runners ahead of time to educate them about the risks of competing in hot weather. In addition, on race day the announcer could remind runners of the WBGT flag level and flags could be used at various distance markers to remind runners of heat hazards. Other races like the Boston Marathon use product like Everbridge (https://www.everbridge.com/) to push weather hazard warnings as text messages to cell phones.

This is a retrospective study that considered many years of data from the Falmouth Road Race. As such, we did not have detailed health information from race participants (e.g., fitness status, heat-acclimatization status, hydration, or pre-existing medical conditions) that may have influenced the risk for an EHS. A key limitation of our study, then, is that we can only provide an association between race pacing and EHS occurrence but not a cause and effect relationship. Even so, we provide a plausible mechanism linking pacing and EHS, and our findings lend support to the well-established practice of weather-based activity modification.

For future work, we hope to add morphological characteristics in our EHS risk stratification since the rate of metabolic heat production and dissipation is directly influenced by body mass and relative body surface area (Cramer and Jay, 2015). Furthermore, identifying barriers for adjusting race day strategy (i.e., race pace) by environmental condition will help us identify effective ways to disseminate safety information that can help modify runners' behavior during warm race.

## Data Availability Statement

The datasets generated for this study are available on request to the corresponding author.

## Ethics Statement

The studies involving human participants were reviewed and approved by University of Connecticut Institutional Review Board. Written informed consent from the participants' legal guardian/next of kin was not required to participate in this study in accordance with the national legislation and the institutional requirements.

## Author Contributions

YH, AG, and DC conceived of the presented idea. AG, YH, DC, JJ, and RS contributed to the design and implementation of the research, to the analysis of the results, and to the writing of the manuscript.

### Conflict of Interest

Falmouth Road Race, Inc. has donated money to the Korey Stringer Institute but not specifically to conduct this study. Falmouth Road Race, Inc. had no influence over the findings or conclusions of the research. The authors declare that the research was conducted in the absence of any commercial or financial relationships that could be construed as a potential conflict of interest.
